# Combinatorial Drug Testing in 3D Microtumors Derived from GBM Patient-Derived Xenografts Reveals Cytotoxic Synergy in Pharmacokinomics-informed Pathway Interactions

**DOI:** 10.1038/s41598-018-26840-4

**Published:** 2018-05-30

**Authors:** Ashley N. Gilbert, Joshua C. Anderson, Christine W. Duarte, Rachael S. Shevin, Catherine P. Langford, Raj Singh, G. Yancey Gillespie, Christopher D. Willey

**Affiliations:** 1 The University of Alabama at Birmingham, Department of Biomedical Engineering, Birmingham, AL 35249 USA; 2 The University of Alabama at Birmingham, Department of Radiation Oncology, Birmingham, AL 35249 USA; 3 Maine Medical Center Research Institute, Portland, Maine, 04101 USA; 4 Vivo Biosciences, Inc., Birmingham, AL 35205 USA; 5 Present Address: Institute of Regenerative Medicine, LifeNet Health, Virginia Beach, VA 23453 USA; 6 The University of Alabama at Birmingham, Department of Neurosurgery, Birmingham, AL 35294 USA

## Abstract

Glioblastoma multiforme (GBM), the most common form of primary malignant brain cancer in adults, is a devastating disease for which effective treatment has remained elusive for over 75 years. One reason for the minimal progress during this time is the lack of accurate preclinical models to represent the patient’s tumor’s *in vivo* environment, causing a disconnect in drug therapy effectiveness between the laboratory and clinic. While patient-derived xenografts (PDX’s or xenolines) are excellent human tumor representations, they are not amenable to high throughput testing. Therefore, we developed a miniaturized xenoline system (microtumors) for drug testing. Nineteen GBM xenolines were profiled for global kinase (kinomic) activity revealing actionable kinase targets associated with intracranial tumor growth rate. Kinase inhibitors for these targets (WP1066, selumetinib, crizotinib, and cediranib) were selected for single and combination therapy using a fully human-derived three-dimensional (3D) microtumor model of GBM xenoline cells embedded in HuBiogel for subsequent molecular and phenotype assays. GBM microtumors closely resembled orthotopically-implanted tumors based on immunohistochemical analysis and displayed kinomic and morphological diversity. Drug response testing could be reproducibly performed in a 96-well format identifying several synergistic combinations. Our findings indicate that 3D microtumors can provide a suitable high-throughput model for combination drug testing.

## Introduction

According to the World Health Organization, Glioblastoma multiforme (GBM) is the most common form of primary malignant brain cancer in adults with a 5-year survival rate of 4–5%^1^. Despite 75 years of research, the projected survival with treatment is only 15 months after diagnosis. Many promising preclinical therapies for GBM have failed to fulfill expectations in subsequent clinical trials, indicating methods for determining drug efficacy are inadequate^1–4^. This disconnect in therapy performance between the laboratory model and the patient is likely due to the fact that most traditional preclinical models, specifically two-dimensional (2D) immortalized cell lines, are poor representations of the human disease and do not accurately recapitulate the *in vivo* environment^2,4–7^. Although patient-derived xenografts (PDX), in which patient tumors are serially passaged in immunocompromised mice, are more attractive model systems of human disease due to the higher preservation (in terms of the tumor heterogeneity, histological, molecular, and genetic characteristics) of the primary tumor, high throughput drug screenings in orthotopic GBM PDXs are not practical due to cost and time limitations^8–11^.

Due to constraints for GBM therapeutic testing in mice, there has been particular interest in producing *in vitro* cell-based culture systems that can combine the primary tumor-like characteristics of the PDXs with the ease of more traditional cell culture^12^. Recently, 3D culture systems have gained increasing recognition as an effective tool for biologic research and high throughput drug testing^13^. These models are unique as they better represent the *in vivo* disease by aiming to restore the 3D architecture that characterizes normal tissues and solid tumors alike^14–16^. Although useful, current 3D systems like microcarrier beads, bioreactors, and cellular spheroids pose many limitations. Some are difficult to adapt for automated imaging and high throughput analysis due to poor reproducibility, and most 3D matrices these models are propagated in, including growth factor-reduced Matrigel, contain intrinsic growth factors and lack important stromal collagens, creating a more artificial environment^17,18^.

To overcome these challenges, we have developed a novel solution in a unique 3D environment that potentially addresses the issues of current preclinical modeling. Using single cells derived from disaggregated athymic nude mice PDXs, we investigated the use of a HuBiogel-based (Vivo Biosciences, Inc., Birmingham, AL; now owned by LifeNet Health, Inc., Virginia Beach, VA) microtumor model system as a drug screening approach for GBM. This natural HuBiogel matrix is derived from discarded human amnion tissue with essential proteins like laminin, collagen I, collagen IV, entactin, tenascin, fibronectin, and proteoglycans^19^. Since HuBiogel is neither angiogenic nor mitogenic, as it lacks all major known growth factors, PDX cells are less subject to growth factor driven selection pressure^20^. Therefore, we hypothesized that this 3D microtumor system would better recapitulate *in vivo* PDX molecular signaling and tumor growth while providing a high throughput assay system for small molecule kinase inhibitor (SMI) combination screening and efficacy testing.

## Results

### Kinomic Profiling of GBM PDX Reveals Potential Actionable Targets

**Figure 1:**
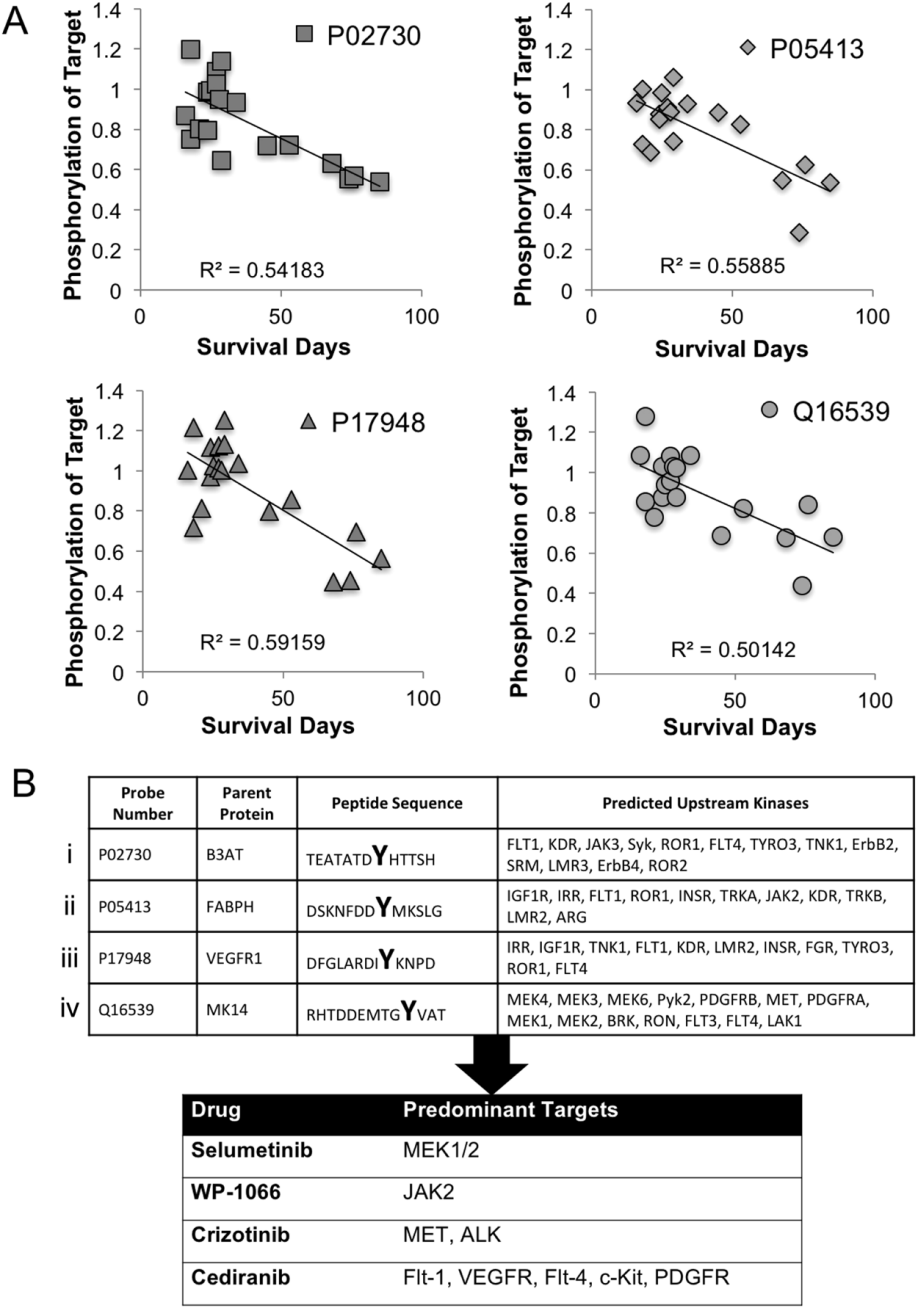
Kinomic profiling of GBM xenolines identify actionable kinase targets for testing. **(A)** Kinomic probes for showing inverse correlation between phospho-peptide intensity and intracranial survival in days for 19 GBM PDX. Correlation (R^2^) is indicated for each peptide probe. **(B)** Probe Number (UniProt ID) and corresponding phosphorylatable peptide sequence with tyrosine residue indicated with bold large font are shown. Predicted upstream kinases for phosphopeptide sequences are indicated (See Materials and Methods for upstream kinase identification strategy). Selected drugs for subsequent characterization are shown with predominant kinase targets.

**Figure 2:**
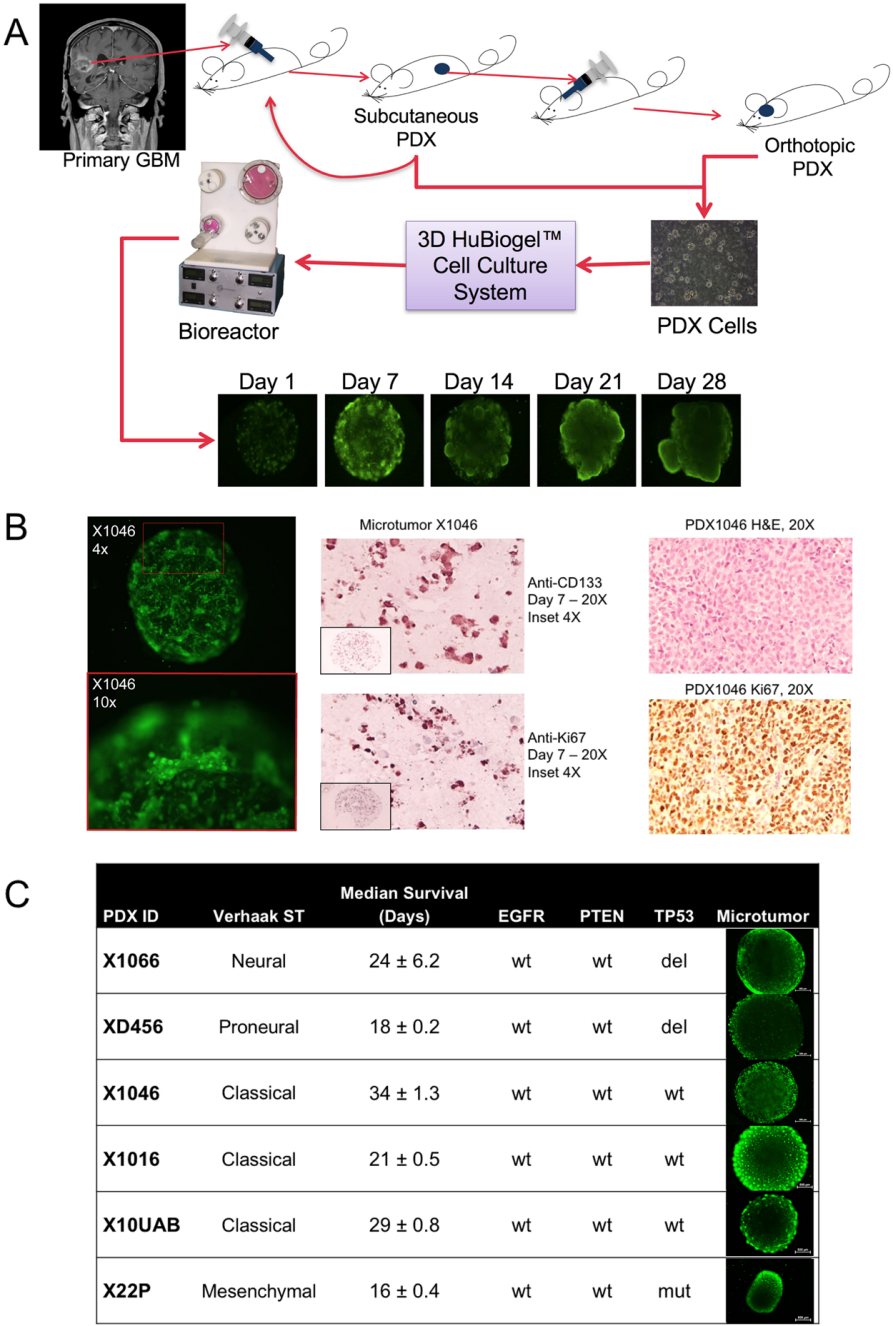
3D xenoline microtumors as model of GBM. **(A)** Schematic for 3D microtumor production from PDX tumor cells. **(B)** Microtumor Calcein-AM live cell staining and IHC compared to PDX tumor. Microtumor characterization using Calcein-AM live cell staining (left 2 panels) and IHC staining of stem cell (CD-133) and proliferation (Ki-67) markers (middle two panels) is shown at low and high magnification to mimic *in vivo* tumors (H&E and Ki-67 staining shown for murine implanted tumors (right two panels) (magnification is indicated). **(C)** Selected PDX tumors with Verhaak *et al*. molecular subtype^40^, median survival, EGFR, PTEN and TP53 status as well as Calcein-AM imaging of Microtumor beads in Neurobasal medium at Day 7 at 4x magnification and 250 ms exposure. Scale bar is 500 µm.

The UAB Brain Tumor Animal Models (BTAM) Core has developed and maintained over 40 GBM PDX tumors. An initial cohort of 19 GBM PDX were kinomically profiled using the PamStation12 peptide substrate microarray platform (protein tyrosine kinase, or PTK PamChip) and phosphopeptide probe intensities were plotted versus PDX survival when implanted intracranially. We identified 4 peptides whose phosphorylation intensity correlated with intracranial growth rate (R^2^ > 0.5). The kinomic activity for these peptides plotted against survival in days for each tumor is shown in Fig. 1A. The peptide sequence and phosphosite for each peptide are shown in Fig. 1B with corresponding upstream kinase specificity based on phosphonet.ca prediction algorithm. As such, we identified 4 small molecule inhibitors (SMI’s) that targeted one or more of the actionable kinase targets that have prior evidence for blood brain barrier penetration. We then wanted to test these SMI’s alone or in combination in GBM PDX. However, due to the inherent cost and throughput constraints of *in vivo* PDX models, we sought to develop a patient-derived model system that replicates key features of an *in vivo* PDX but in a high throughput testing format. As such, we utilized a three-dimensional (3D) microtumor system produced by embedding GBM PDX cells in a fully human matrix material (HuBiogel)^21^ shown schematically in Fig. 2A.

### Establishment of GBM Microtumors and Morphological Assessment

PDX tumor cells derived from tumors grown subcutaneously in athymic nude mice were prepared as single cell suspensions and grown as microtumors (See Methods for details). IHC analysis and Calcein-AM live cell imaging were performed to characterize the microtumors with determination of general morphology of the cells inside the microtumors and to confirm viability. Hematoxylin and eosin, Ki-67, and CD133 staining of microtumor cross-sections demonstrate preservation of proliferative capacity, colony-like formation, and invasion processes during long-term culture as compared to matching murine-implanted tumors(Fig. 2B). Subsequent experiments were performed in a subset of six PDX lines with molecular characteristics, median intracranial survival information, and microtumor morphology (Calcein AM stained cells) shown in Fig. 2C. In terms of microtumor morphological characterization, all PDX microtumors started out at Day 1 with viable cells that were uniformly dispersed throughout the matrix. By Day 7, however, we observed two different microtumor morphologies: (1) a nodular growth pattern in JX10UAB, XD456, and X1016 and (2) diffuse spreading in JX22P, X1066, and X1046 (Fig. 2C). Interestingly, we also visualized microtumor bead contraction in the JX22P xenoline (Fig. 2C). This contraction was specific to this xenoline and very reproducible. It should be noted that JX22P was the one PDX of Mesenchymal subtype (Verhaak *et al*. classification).

### Kinomic Profiles Reveal Microtumors Cluster Together by Tumor Type

**Figure 3:**
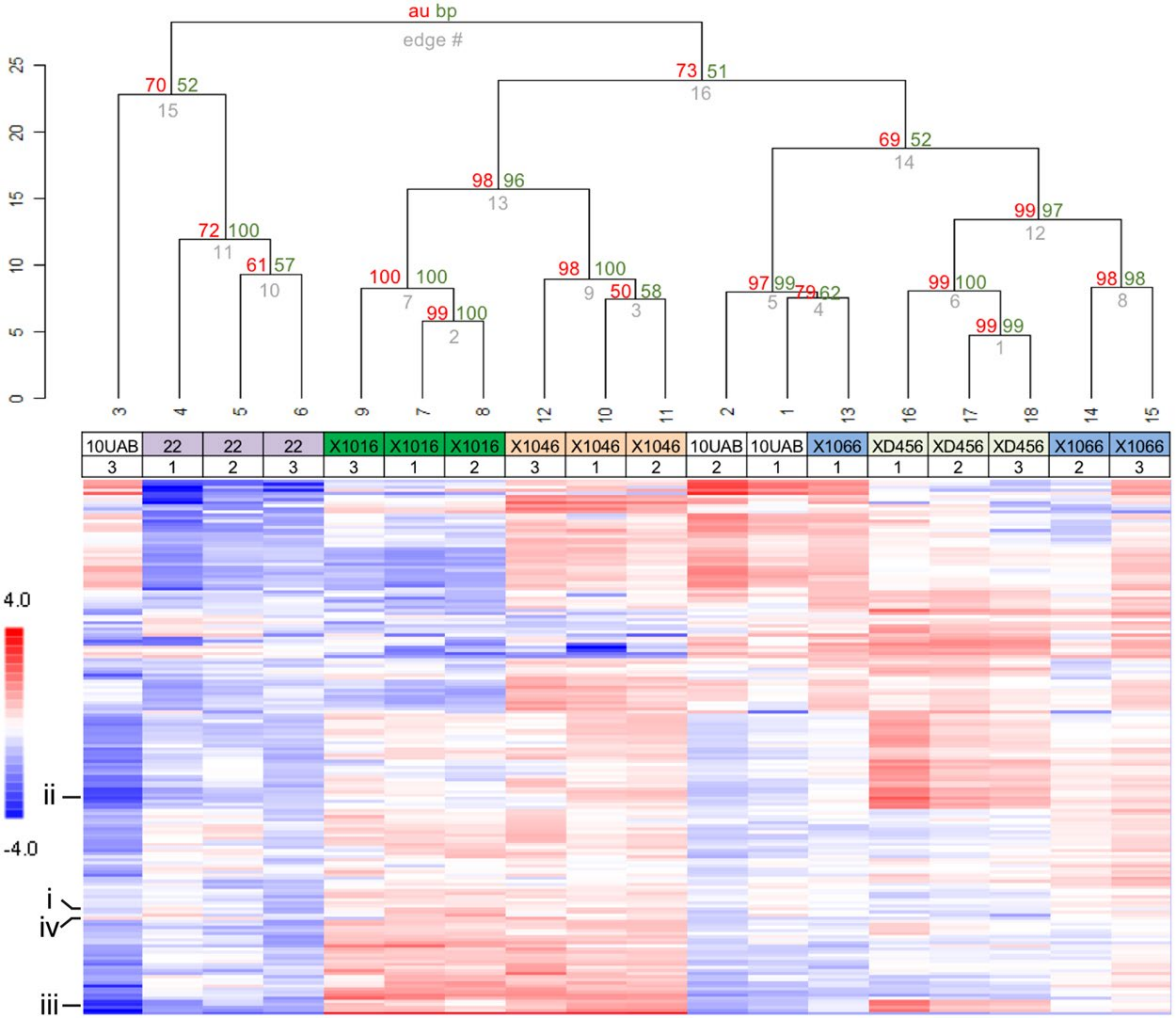
Kinomic profiling showing molecular diversity of GBM xenoline microtumors. Kinomic heatmap of combined PTK and STK chip analyses with triplicates of each microtumor xenoline at Day 7. The GBM xenoline identity is labeled with biological replicate number indicated below the xenoline. Each cell is a phosphopeptide probe displayed as log-transformed change from peptide mean as higher (red) or lower (blue) than the average signal per peptide. Bootstrap resampling probability testing of clustering robustness using pvclust (R script). approximately unbiased (AU) score is shown in red and a normal bootstrap probability value (BP) is shown in green at the dendrogram hinges. AU values ≥95 indicate that the data highly supports the clustering. The 4 peptides shown in Fig. 1B are indicated.

Having demonstrated that the GBM microtumors could replicate many of the features seen in intact tumors, we next sought to determine whether the PDX microtumors demonstrated biological diversity by examining their kinase signaling. For this, we performed global kinase activity (kinomic) assessments of the PDX microtumors using the PamStation12 platform technology (Fig. 3). Initial kinomic profiling of the replicate microtumors using an unsupervised hierarchical clustering approach is shown in the heatmap with associated dendrogram, where samples are clustered by column (samples) and row (peptides) using a geometric distance-means based clustering, where the height of the vertical lines on the dendrogram represent the degree of similarity in the cluster. Bootstrap probability metrics for the clustering were generated using the R script pvclust^22,23^. The AU and BP scores are displayed on the dendrogram in red and green, respectively, indicating that the data supports the clustering. Based on our results, we determined that each PDX has a relatively distinct kinomic profile and that microtumor replicates retain similar kinomic profiles, though a single replicate of JX10UAB and X1066 had deviations from their other replicates that affected their clustering.

### 3D Microtumor Drug Screen

**Figure 4:**
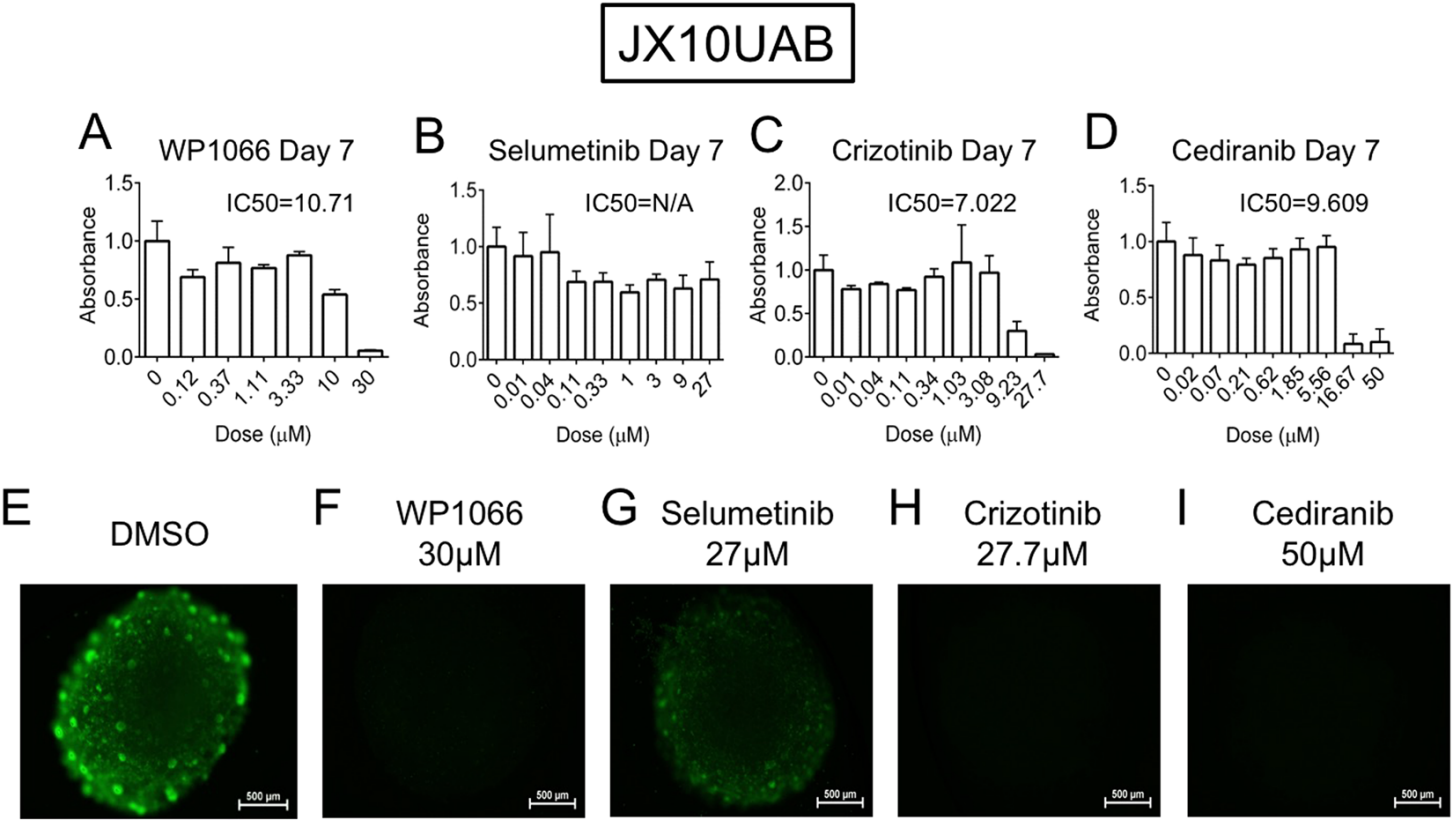
Dose response of JX10UAB to WP1066, Selumetinib, Crizotinib, and Cediranib at Day 7. Raw MTT absorbance of **(A)** WP1066, **(B)** Selumetinib, **(C)** Crizotinib and **(D)** Cediranib are shown with DMSO control representing the 0 µM dose with calculated IC50 indicated (N/A = not applicable). **(E**–**I)** Calcein-AM imaging of DMSO or highest doses of each drug (30 µM, 27 µM, 27.7 µM, and 50 µM, respectively) at Day 7 at 4x magnification and 250 ms exposure. Scale bar is 500 µm.

Having identified that the PDX microtumors maintain kinomic diversity with biological replicate reproducibility, we optimized a drug response assay through initial dose response studies using the four small molecule inhibitors (Fig. 1B): WP1066, selumetinib, crizotinib, and cediranib. All of the microtumor xenolines demonstrated sensitivity to WP1066, crizotinib, and cediranib based on MTT assay (Supplementary Fig. S1) and decrease in fluorescence (Calcein-AM) (Supplementary Fig. S2), at the highest doses of each, 30 µM, 27 µM, and 50 µM, respectively, with the JX10UAB xenoline shown as an example (Fig. 4 and Supplementary Fig. S3). We noted marked cytotoxicity across a broad range of xenolines with cediranib and crizotinib. WP1066 sensitivity was more variable in our xenoline cohort. Nonetheless, IC_50_s for WP1066, crizotinib, and cediranib could be calculated for the xenolines. However, selumetinib, at the highest dose of 27 µM, induced little, if any, cytotoxicity in all xenolines (Supplementary Fig. S1 and S2). As such, we concluded that the xenolines were generally insensitive to selumetinib monotherapy and a dose titration curve could not be generated. Accordingly, we selected an arbitrary “IC_50_^*^” of 5 µM for selumetinib based on the published IC_50_ range for selumetinib and the IC_50_s of the other three SMIs in order to utilize selumetinib in the combination treatment assay described below. IC_50_ means and standard deviations obtained from the Day 7 dose finding data for the remaining three SMIs were as follows: WP1066, 8.71 ± 2.92 µM; crizotinib, 6.39 ± 2.71 µM; and cediranib, 6.41 ± 2.36 µM. IC_37.5_, IC_25_, and IC_12.5_ doses were calculated from this data.

### Synergy Testing of SMIs in microtumors

**Figure 5:**
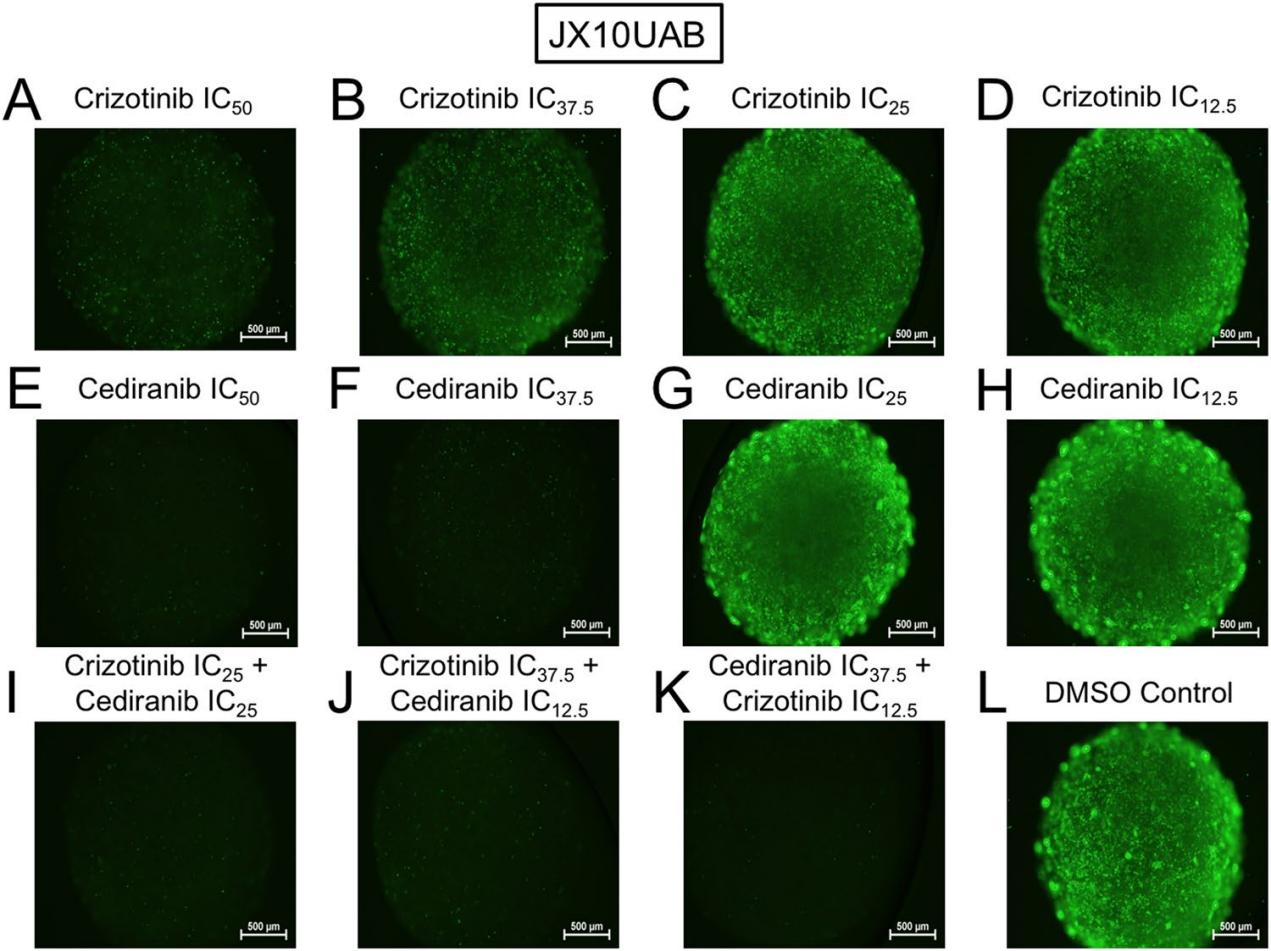
Calcein-AM imaging of drug combinations in GBM xenoline microtumors. Calcein-AM imaging of **(A**–**H)** single and **(I**–**K)** combination drug screening of Crizotinib and Cediranib and (**L**) DMSO control of the JX10UAB xenoline at Day 7 at 4x magnification and 250 ms exposure. Scale bar is 500 µm.

Following the single drug dose finding studies, we pursued combination testing of the four SMI’s in each of the PDX microtumors. Drug interaction assignment (synergism versus additive versus antagonism) was determined by CI calculation of the MTT viability data using CalcuSyn software and is displayed in Table 1 and Supplemental Table S1. Overall, out of a total of 30 synergistic combinations across all cell lines, WP1066 was present in 8 combinations, selumetinib was present in 14 combinations, crizotinib was present in 20 combinations, and cediranib was present in 18 combinations. Crizotinib and cediranib made the greatest number of appearances in synergistic combinations alone, as well as in combination together, compared to the other SMIs and combinations. This was not limited to only 1 or 2 xenolines as crizotinib and cediranib were the most commonly represented in synergistic combinations across all xenolines. For illustration purposes, JX10UAB xenoline microtumors with Calcein-AM imaging are shown for combinations of crizotinib and cediranib to demonstrate the unique morphology and overall cytotoxicity observed (Fig. 5). WP1066 was not part of synergistic combinations in X1066, X1016, or X1046 xenolines. Selumetinib was not part of synergistic combinations in XD456 or X1046. Crizotinib was not part of synergistic combinations in X1016. However, cediranib was part of synergistic combinations in all xenolines.

**Table 1:** Synergistic combination index (CI) values of WP1066, Selumetinib, Crizotinib, and Cediranib SMIs in combination for all xenolines at Day 7.

Xenoline	Combination Dosing	CI	Description
XD456			
	Cediranib IC_37.5_ + Crizotinib IC_12.5_	0.76	Synergism
	Crizotinib IC_37.5_ + WP1066 IC_12.5_	0.80	Synergism
	Crizotinib IC_25 + _Cediranib IC_25_	0.85	Synergism
X1066			
	Cediranib IC_37.5_ + Selumetinib IC_12.5_	0.71	Synergism
	Selumetinib IC_25 + _Cediranib IC_25_	0.72	Synergism
	Cediranib IC_37.5_ + Crizotinib IC_12.5_	0.96	Synergism
	Crizotinib IC_37.5_ + Selumetinib IC_12.5_	0.97	Synergism
JX10UAB			
	Crizotinib IC_37.5_ + Selumetinib IC_12.5_	0.57	Synergism
	Cediranib IC_37.5_ + Selumetinib IC_12.5_	0.75	Synergism
	Selumetinib IC_25 + _Crizotinib IC_25_	0.81	Synergism
	WP1066 IC_37.5_ + Selumetinib IC_12.5_	0.91	Synergism
	Cediranib IC_37.5_ + Crizotinib IC_12.5_	0.93	Synergism
	Crizotinib IC_37.5_ + Cediranib IC_12.5_	0.95	Synergism
JX22P			
	Crizotinib IC_37.5_ + Selumetinib IC_12.5_	0.08	Synergism
	Crizotinib IC_37.5_ + WP1066 IC_12.5_	0.15	Synergism
	Selumetinib IC_25 + _Crizotinib IC_25_	0.22	Synergism
	Crizotinib IC_37.5_ + Cediranib IC_12.5_	0.28	Synergism
	WP1066 IC_37.5_ + Selumetinib IC_12.5_	0.31	Synergism
	WP1066 IC_37.5_ + Crizotinib IC_12.5_	0.47	Synergism
	Selumetinib IC_25 + _Cediranib IC_25_	0.60	Synergism
	Crizotinib IC_25 + _Cediranib IC_25_	0.61	Synergism
	WP1066 IC_37.5_ + Cediranib IC_12.5_	0.63	Synergism
	Cediranib IC_37.5_ + Selumetinib IC_12.5_	0.67	Synergism
	Selumetinib IC_37.5_ + Crizotinib IC_12.5_	0.70	Synergism
	Cediranib IC_37.5_ + Crizotinib IC_12.5_	0.79	Synergism
	WP1066 IC_25 + _Crizotinib IC_25_	0.86	Synergism
	WP1066 IC_25 + _Cediranib IC_25_	0.96	Synergism
X1046			
	Crizotinib IC_25 + _Cediranib IC_25_	0.08	Synergism
	Cediranib IC_37.5_ + Crizotinib IC_12.5_	0.09	Synergism
X1016			
	Selumetinib IC_37.5_ + Cediranib IC_12.5_	0.12	Synergism

## Discussion

GBM is a devastating disease for which effective treatment has remained elusive. It is theorized that one of the main reasons for this is the lack of accurate and reliable preclinical models that represent the *in vivo* environment, which causes a disconnect in therapy effectiveness between the laboratory and clinic. Our proposed solution to address this disconnect and the issues of traditional and current preclinical modeling is to enlist the use of innovative HuBiogel-based microtumors as a 3D drug screening model for GBM. We demonstrate that the microtumor model has promising potential as a complementary model for the more gold standard orthotopic PDX model system.

In a recent overview, the HuBiogel-based *in vitro* Microtumor system was determined to be more reflective of the mouse *in vivo* environment in comparison to a 2D plate culture model when studying major kinase signaling networks (e.g., expression profile of ERK, pERK, CRAF, and KIT targets)^24^. The 3D cultured microtumors displayed unique kinomic profiles (Fig. 3) suggesting that the HuBiogel environment allows for signaling diversity for implanted tumor cells. In our prior microtumor methodology publication^21^, we detected kinomic changes in JX10UAB microtumors following treatment with temozolomide, the current clinical standard of care chemotherapy suggesting that the model system could be useful for investigating mechanisms of sensitivity/resistance to therapeutic agents due to the physiological relevance of 3D tumors coupled with molecular profiling.

While observing Calcein-AM imaging data, we found that JX10UAB, X1016, and XD456 xenolines formed sphere-like nodules within the HuBiogel matrix throughout all experiments, potentially due to the stem-like cells generally seen in neurosphere culture as GBM PDXs have previously been successfully cultured in this manner^25–27^. These neurosphere-forming cells are often termed ‘brain tumor stem cells’ or ‘brain tumor initiating cells’ (BTIC’s) because of their capacity to self-renew and differentiate into cell types present within the tumor of origin^25,28^. Recently, our group has shown that BTIC’s are effectively cultured as microtumors and maintain tumorigenicity *in vivo*^29^. Our detection of CD133 positive staining cells in the microtumors (Fig. 2B) lends further support to this notion.

According to the MTT data and imaging from both dose titration and drug screening experiments, WP1066, the JAK2/STAT3 kinase inhibitor, played a large role in both overall cell death in all xenolines and nodule degeneration in the JX10UAB and X1016 xenolines. Indeed, a prior study determined that JAK2/STAT3 inhibition with this SMI dramatically reduced brain tumor stem cell survival and prolonged mouse survival with orthotopic xenografts, and another study demonstrated that WP1066 inhibited the STAT3 pathway, inducing apoptosis in malignant glioma cells both *in vitro* and *in vivo* by down regulating anti-apoptotic proteins^30,31^. Both give possible rationalizations for the cytotoxic effect of WP1066 in GBM PDX microtumors.

Crizotinib, the dual c-MET and ALK receptor tyrosine kinase (RTK) inhibitor, also played a role in cytotoxicity across all xenolines as well as disaggregating the sphere-like nodules in the JX10UAB and X1016 PDX microtumors. In two recent studies using crizotinib, it was determined that this SMI exhibited potent anti-glioma effect in c-MET expressing GBM stem-like cells, consistent with c-MET’s role in maintaining GBM stem-like cell self-renewal capacity *in vitro*, demonstrating a likely hypothesis and mechanism of action for the effectiveness of crizotinib, not only in JX10UAB and X1016, but also in other xenolines as well^32,33^.

In several previous studies, cediranib showed promising results in gliomas, leading to 6-month progression-free survival in phase II clinical trials^34^. The efficacy of cediranib has been related to its anti-angiogenic capability and ability to normalize tumor vasculature and alleviate edema in glioma patients via ATP-competitive inhibition of VEGFR signaling. In addition, cediranib significantly inhibits tyrosine kinase activity for c-Kit, PDGFR-α, and PDGFR-β^2,35,36^. However, *in vitro* testing the effects of cediranib has been limited. One study showed that cediranib had cytotoxic effects, inducing GBM cell death by apoptosis, inhibiting cellular migration and invasion, and further confirmed this by *in vivo* assays showing that cediranib displayed antiangiogenic and antitumoral activity in GBM^3^. Along with our studies, these indicate that cediranib is likely an effective SMI for GBM, though the exact target (both kinase and cell target) promoting these effects is not clearly established.

As our studies have shown, selumetinib, the MEK1/2 kinase inhibitor, showed little effect with regard to cell death as a monotherapy, which is in stark contrast to many studies and clinical trials showing the effectiveness of selumetinib in other cancers^37,38^. However, one group has shown that while MEK1/2 inhibitors decreased levels of phosphorylated-ERK1/2 in GBM, regardless of NF1 status, growth inhibition occurred only in neurofibromin 1 (NF1)-deficient cells^39^, a protein that is frequently mutated in the Mesenchymal subtype^40^. Interestingly, in the CI data for the JX22P xenoline in our research, selumetinib was found in 6 out of 14 synergistic combinations and 18 total combinations, the largest number of synergistic effects with selumetinib of any xenoline, demonstrating that selumetinib may have been most effective in JX22P due to its Mesenchymal subtype. While virtually ineffective for cell death, selumetinib did show some morphological changes in the JX10UAB (Fig. 4G) and X1016 xenolines (Supplementary Fig. S2). The Classical subtype of GBM is recognized by epidermal growth factor (EGFR) amplifications, and the overexpression of EGFR has been associated with elevated levels of phosphorylated ERK1/2, possibly indicating increased levels or activity MEK1/2^41^. Specifically, according to synergy testing, JX10UAB and X1016, both of the Classical subtype, showed synergistic effects with selumetinib; therefore, it is possible that these two xenolines were particularly morphologically sensitive to the SMI.

## Conclusion

In conclusion, we describe a 3D GBM PDX microtumor system using the HuBiogel microenvironment that appears to replicate *in vivo* tumor conditions while maintaining diverse intra-tumoral signaling. Furthermore, this model appears to be suitable for high-throughput drug testing, including combination drug treatment in a cost- and time-effective manner. Future studies comparing 3D HuBiogel microtumor drug testing to traditional PDX animal model studies are needed to ultimately determine its preclinical/translational utility.

## Methods

### Xenolines and Culture Conditions

PDX (also referred to as “xenolines” to distinguish serial mouse passaged tumor cells from immortalized tumor cells passaged in cell culture) passaging and dissociation is a well-established practice within the Brain Tumor Animal Model (BTAM) Core (University of Alabama at Birmingham) and has been previously well described^21,42^. All animal studies were carried out in accordance with the policies set by the UAB Institutional Animal Care and Use Committee and performed according to their guidelines. Moreover, the experimental protocols were registered and approved by UAB Occupational Health & Safety (Project# 14–124). Female athymic nude mice were obtained from Harlan Laboratories. All primary patient tumors used for this study were either obtained from surgical resection at UAB or were a gift from Dr. David James (Mayo Clinic, Rochester, MN) (JX22P and JX10UAB) or obtained from Dr. Darrell Bigner (Duke University) (XD456). All PDXs were serially passaged subcutaneously by harvesting whole tumor from normal tissue, washing with Phosphate Buffered Saline (PBS), mincing with scalpels, and further mechanical scoring into a slurry for subcutaneous injection into the flank. If ready for microtumor production, after mincing, cells were dissociated into single suspension via Miltenyi Tumor Dissociation Kit (Miltenyi Biotec, Inc. Order Number 130-096-730). An initial cohort of 19 xenolines from the UAB Brain Tumor Animal Model (BTAM) core were used for initial kinomic assessment and correlation to survival. For subsequent microtumor studies, we selected for six xenolines for this study: JX10UAB, XD456, X1016, X1046, JX22P, and X1066. Single cell suspensions were generated from subcutaneously implanted PDX tumors in mice in 50 mL of complete Neurobasal medium (Invitrogen Catalog Number 10888-022), containing 1% N-2 Supplement (Invitrogen Catalog Number 17502-048), 2% B-27 Supplement (Invitrogen Catalog Number 12587-010), 10 ng/mL FGF 2 (Invitrogen Catalog Number PHG0266), 10 ng/mL EGF (Invitrogen Catalog Number PHG0315), 1% L-Glutamine (Cellgro Catalog Number 25-005-CI), 1% Penicillin-Strep (Omega Scientific, Inc. Catalog Number PS-20), 1% Fungizone (Omega Scientific, Inc. Catalog Number FG-70), and 0.1% Gentamicin (Invitrogen Catalog Number 15750-060). Cells were transported from the BTAM to Vivo Biosciences, Inc. on wet ice.

### Microtumor Production

Detailed description of microtumor production has been previously described^11,12,21,29^. Briefly, after receiving the disaggregated PDX cells (taken from tumor in athymic nu/nu mice), viable single cells were counted with Trypan blue via the TC10 automated cell counter (Bio-Rad Laboratories, Inc.) and mixed with HuBiogel at an exact ratio (Vivo Biosciences, Inc.) of 1,000-2,000 cells/µL of 3 mg/mL HuBiogel solution at 4 C. Then, using a custom 96-pin steel plate, microtumors were generated from this mixture (Day 0), each containing 50,000 cells and measuring 2 mm in diameter. This was done by rapidly dispensing 10 µL into a plastic dish using a multi-channel pipette and subjected to gelation at 37 C in a tissue culture incubator to form a microtumor bead with total time of less than 15 minutes yielding >90% cell viability. 3D microtumor beads were then transferred to a Synthecon rotary bioreactor (100 mL) and cultured in neurobasal media for 2–3 days to produce uniform 2 mm microtumors. After production, these free-floating microtumors were placed in 96-well tissue culture plates in the aforementioned complete Neurobasal medium with one microtumor per well. Cells were incubated at 37 °C in 5%, humidified CO_2_.

### Immunohistochemical Analysis of Microtumors

Immunohistochemical (IHC) analysis was performed using the Super Sensitive Polymer-HRP IHC Detection System (Biogenex) according to manufacturer’s protocol with minor exceptions. Briefly, microtumors were prepared at day 7 for immunohistochemical (IHC) staining by pre-staining them with hematoxylin for 1 min followed by a calcium and magnesium supplemented phosphate buffered saline (PBS) rinse to facilitate microtumor visualization. The tumors are then placed in tissue cassettes for paraffin embedding. For IHC, samples were deparaffinized using citrisolv, isopropanol, and water followed by antigen retrieval of slides using citrate buffer (DAKO) and steamer. Slides were blocked using 3% hydrogen peroxide in methanol for 3 min at room temperature followed by PBS rinse and then a peroxidase block (Biogenex kit) for 10 min also at room temperature. Slides are subject to power block solution (Biogenex) for 20 minutes at room temperature and then gently blotted. Primary antibody is then applied to each slide in PBS-1%BSA for 1 hour at room temperature. Following 5 × 5 min PBS rinses, Super Enhancer is applied for 20 min at room temperature followed by 4 × 5 min PBS washes. Secondary antibody (Biogenex kit) is applied for 30 min at room temperature followed by 3 × 5 min washes. Detection and counterstaining is performed using DAB substrate (Biogenex kit) followed by hematoxylin staining, ethanol/xylene treatment and mounting of coverslip. Primary antibodies and dilutions were as follows: ki-67 (1:75, DAKO), and Prominin-1 (CD133) (1:200, BiorByt).

### Dose Titration of Microtumors

Serial dilutions of four small molecule inhibitors (SMIs), WP1066 (inhibiting JAK2/STAT3), selumetinib (inhibiting MEK1/2), crizotinib (inhibiting c-MET and ALK), and cediranib (inhibiting VEGFR, Flt-1, Flt-4, c-Kit, and PDGFR) (Selleckchem, Houston, TX, Catalog Numbers S2796, S1008, S1068, S1017, respectively), along with the vehicle control (dimethyl sulfoxide or DMSO) were used to establish a dose titration curve for determining appropriate concentrations of drugs required for 50% inhibition *in vitro* (IC_50_s) in the 3D microtumor model. All dose-finding experiments were performed as 3-fold serial dilutions, 0.5% final DMSO in triplicate. High and/or low doses were selected based on the literature and preliminary experiments^3,31,43–46^. High doses were as follows: WP1066, 30 µM; selumetinib, 27 µM; crizotinib, 3 µM (for X1046 and X1016) and 27 µM (for XD456, JX10, X1066, and JX22P); and cediranib 50 µM. Drugs or vehicle were introduced on Day 1 and were reintroduced every three days when media was changed. For media changing, old media was completely removed, and fresh complete Neurobasal® media and drug dosing solutions were added.

### Cell Viability and Analysis for Dose Titration Experiments

Cell viability was analyzed 6 days after initial treatment (Day 7) using the 3-(4,5-dimethylthiazol-2-yl)-2,5-diphenyltetrazolium bromide (MTT) colorimetric assay (LifeTechnologies, Catalog Number M6494) similar to our prior studies^21,29^. Briefly, microtumors were transferred to 96-well plates using an electronic dispenser with wide-mouth pipet tips (ViaFlo) to ensure that each well in the 96-well plate contained one microtumor bead, and each condition had at least three replicates. MTT was prepared, and the manufacturer’s protocol was followed for lysis, dissolution, and analysis, measuring absorbance at 570 nm. Raw MTT absorbance data was obtained, and average background control was subtracted from raw data using a Synergy HT fluorescence plate reader (Biotek). Dose response data was analyzed using GraphPad Prism version 4 (GraphPad Software, San Diego, CA), and dose response curves were assembled by normalizing the absorbance to DMSO control and plotted as bar graphs. From the dose finding experiments and acquired dose response curves, the IC_50_ means and standard deviations were calculated via GraphPad Prism using non-linear regression curve fitting approach. Dose titration curves and raw data were used to determine the average IC_50_s for each drug, excluding selumetinib for which an “IC_50_*” dose was selected (due to low efficacy, true IC_50_ could not be determined), for subsequent single and combination drug screening.

### Single and Combination Screening in Microtumors

Single and combination drug studies were carried out and repeated with biological quadruplicates. Based on the average IC_50_s for each drug, IC_37.5_s, IC_25_s, and IC_12.5_s for each SMI were calculated. For single drug studies, cells were dosed with an IC_50_, IC_37.5_, IC_25_, and IC_12.5_ of each drug separately for a total of 16 single doses. For combination drugs studies, cells were dosed with IC_25_ + IC_25_ and IC_12.5_ + IC_37.5_ for each of the 4 drugs for a total of 18 combinations on 96 well plates. For all drug dosing experiments, outlying replicates were removed using the Grubbs’ test (two-sided α = 0.5), and for dose finding experiments, the ROUT method was used to identify and remove outlying dose response points in dose titration curves (Q = 1%)^47^.

### Cell Viability and Analysis for Single and Combination Drug Screening

Cell viability was analyzed on Day 7 using the MTT assay as described earlier. Day 7 response data and synergy for single and combination drug studies were evaluated using the Chou-Talalay method of synergy testing via CalcuSyn^48^. In Microsoft Excel, the raw MTT absorbance data from quadruplicate samples were normalized to the DMSO control. Then, to determine effect, normalized absorbances were subtracted from 1. The quadruplicate sample values were then averaged together to obtain a mean. These effect data were entered into CalcuSyn (v. 2.1) as single doses and combinations (non-constant ratios). Combination Indices (CI) were generated. CI values and their indications are as follows: <1 is synergistic, =1 is additive, and >1 is antagonistic. In cases where neither single nor combination dosing resulted in an inhibitory effect less than 25% of control levels, CI scores were not determined and reported as “not a number” (NaN)^49^.

### Calcein-AM Imaging of Microtumors

Calcein-AM (LifeTechnologies, Catalog Number C3099) was used to image the cells inside the microtumors during dose titration and drug screening as a validation approach. The Calcein-AM assay was carried out according to manufacturer’s protocol for Day 7 time points when possible. Images were then acquired with Nikon NIS-Element imaging software via Nikon Eclipse TS100 inverted fluorescent microscope, a Nikon B-2A FITC filter block (λ_excitation_ 465–495 nm bandpass, dichroic mirror 500 nm, λ_emission_ 500 nm long pass), EXFO X-Cite series 120 fluorescent source, and Q Imaging QICAM 12-bit Color Fast 1394 camera.

### Kinomic Profiling and Analysis of Microtumors

Direct measurement and identification of intrinsic kinase activity in GBM xenolines as well as GBM PDX microtumors was determined using kinomic profiling and analysis. Nineteen GBM xenolines were implanted into the brains of athymic nu/nu mice and once established, mice were sacrificed and tumors were harvested and macrodissected to produce total protein lysates for kinomic testing as previously described^50^. For microtumor tissue lysate preparation, two microtumor beads of each xenoline, in biological triplicate at Day 7, were lysed in pre-chilled MPER lysis buffer (Pierce, ThermoScientific, Catalog Number 78501), mixed with a 1:100 ratio each of Halt’s Protein Phosphatase Inhibitor cocktail (Pierce, ThermoScientific, Catalog Number 78420) and Halt’s Protein Protease Inhibitor (Pierce, ThermoScientific, Catalog Number 87786) according to manufacturer’s protocol. Protein quantification for each sample was performed with the BCA Protein Assay Kit (Pierce, ThermoScientific Catalog Number 23225) also according to manufacturer’s protocol. Kinome analysis was performed on orthotopic protein lysates and Day 7 microtumor lysates using the PamStation®12 (PamGene International, The Netherlands) in the UAB Kinome Core (www.kinomecore.com). For kinomic analysis on the tyrosine kinase (PTK) microarray platform, 15 μg of protein were loaded for each sample onto the PamChip, and for the analysis on the serine-threonine kinase (STK) microarray platform, 2 μg of protein of each sample were loaded onto the PamChip. Kinomic signaling was analyzed using Evolve 2 (PamGene) for initial sample and array processing as well as image capture. BioNavigator (v. 6, PamGene) was used for raw data transformation into kinetic (“initial velocity”) values and steady state (“postwash”) values. Postwash values, which were the slopes of 10, 20, 50, 100, and 200 ms exposures multiplied by 100 and transformed by log base-2, were exported. Then, in BioNavigator, samples were clustered together using Euclidian distance means-based hierarchal clustering method (on both columns and rows) with complete linkage through R script. To confirm the clustering, a secondary analysis was performed using pvClust R script^23^, which clusters all non-zero peptides, as we have done previously^22^. A “p-value” is generated for the dendrogram branches using a multiscale bootstrap resampling approach to generate an “approximately unbiased” (AU) score and a normal bootstrap probability value (BP). AU ≥ 95 was considered to be highly supported by the data.

For supervised analysis, kinomic probes for the 19 orthotopic GBM xenolines were plotted versus the median survival of intracranial tumor-bearing mice. Peptides with >0.5 R^2^ correlations were selected for subsequent analysis of upstream kinase prediction using the phosphonet.ca database. Highly ranked upstream kinases were identified as actionable kinase targets.

### Biostatistics

Statistical testing was performed using Graphpad Prism, Calcusyn, and R scripts as described in the individual assay sections.

### Data Availability

The data generated during this study are included in this published article (and its Supplementary Information Files). The kinomic datasets generated during the current study are available from the corresponding author on reasonable request.

## Electronic supplementary material

Supplementary Data
