# Supplementary material for: Combinatorial Drug Testing in 3D Microtumors Derived from GBM Patient-Derived Xenografts Reveals Cytotoxic Synergy in Pharmacokinomics-informed Pathway Interactions
Source: Sci Rep. 2018 May 30;8:8412. doi: 10.1038/s41598-018-26840-4 (PMC5976646; doi:10.1038/s41598-018-26840-4)
Supplement: Supplementary file 1 — Supplementary Data [file 41598_2018_26840_MOESM1_ESM.pdf]

Supplemental Data For:

Combinatorial Drug Testing in 3D Microtumors Derived from GBM Patient-Derived Xenografts Reveals Cytotoxic Synergy in Pharmacokinomics-informed Pathway Interactions

Ashley N. Gilbert,[1] Joshua C. Anderson,[2] Christine W. Duarte,[3] Rachael S. Shevin,[4] Catherine P. Langford,[5] Raj Singh,[4] G. Yancey Gillespie,[5] Christopher D. Willey,[1,2]

[1], Department of Biomedical Engineering, [2], Department of Radiation Oncology, The University of Alabama at Birmingham, [3], Maine Medical Center Research Institute, Portland, Maine, [4], Vivo Biosciences, Inc., Birmingham, Alabama, [5], Department of Neurosurgery, The University of Alabama at Birmingham

Supplemental Figure Captions

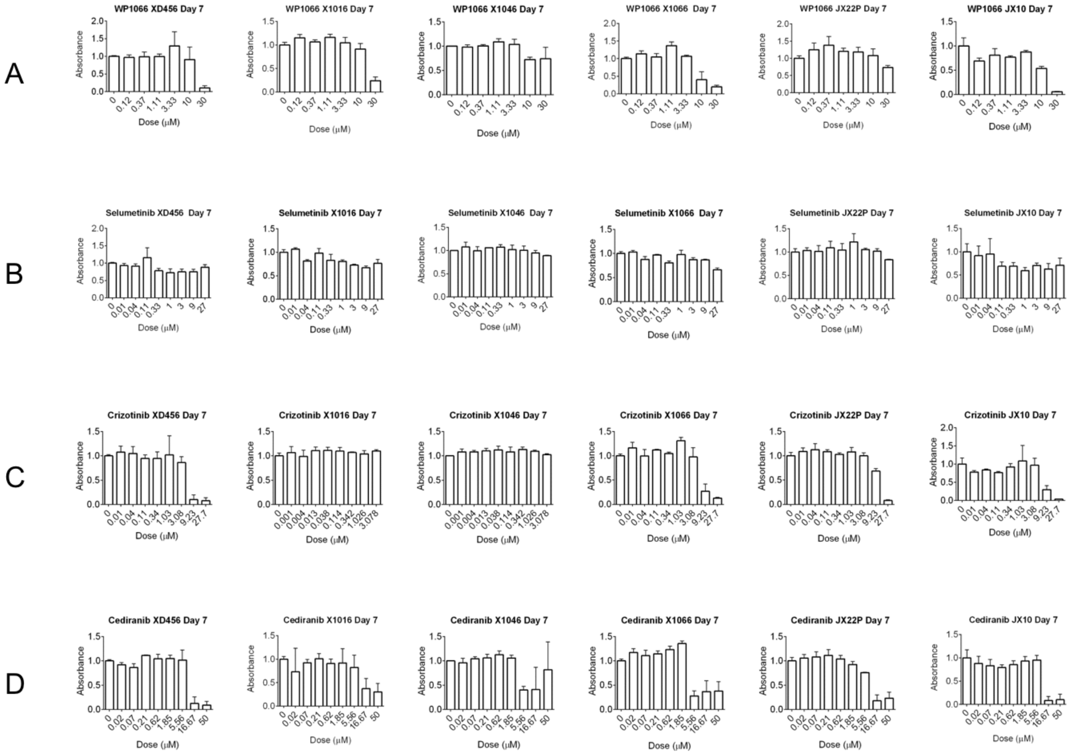

Supplemental Figure S1. Dose response of XD456, X1016, X1046, X1066, and JX22P to WP1066, Selumetinib, Crizotinib, and Cediranib at Day 7. (A-D) Raw MTT absorbance of WP1066, Selumetinib, Crizotinib, and Cediranib, respectively, across xenolines. DMSO control is at 0μM, and dosages increase to the right.

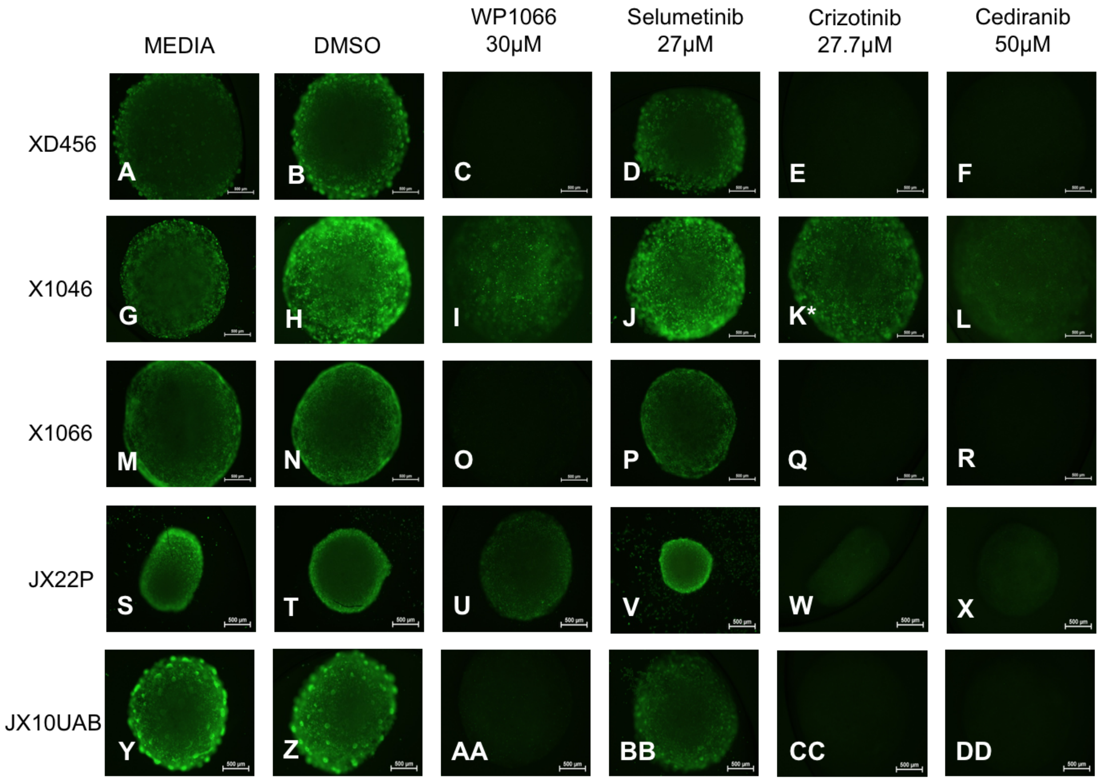

Supplemental Figure S2. Calcein-AM imaging of drug monotherapy in GBM xenoline microtumors. Calcein-AM imaging of **(A-F)** XD456, **(G-L)** X1046, **(M-R)** X1066, **(S-X)** JX22P, and **(Y-DD)** JX10UAB at Day 7 following treatment with WP1066, Selumetinib, Crizotinib, and Cediranib at the indicated concentrations at 4x magnification and 250ms exposure. Scale bar is 500μm.

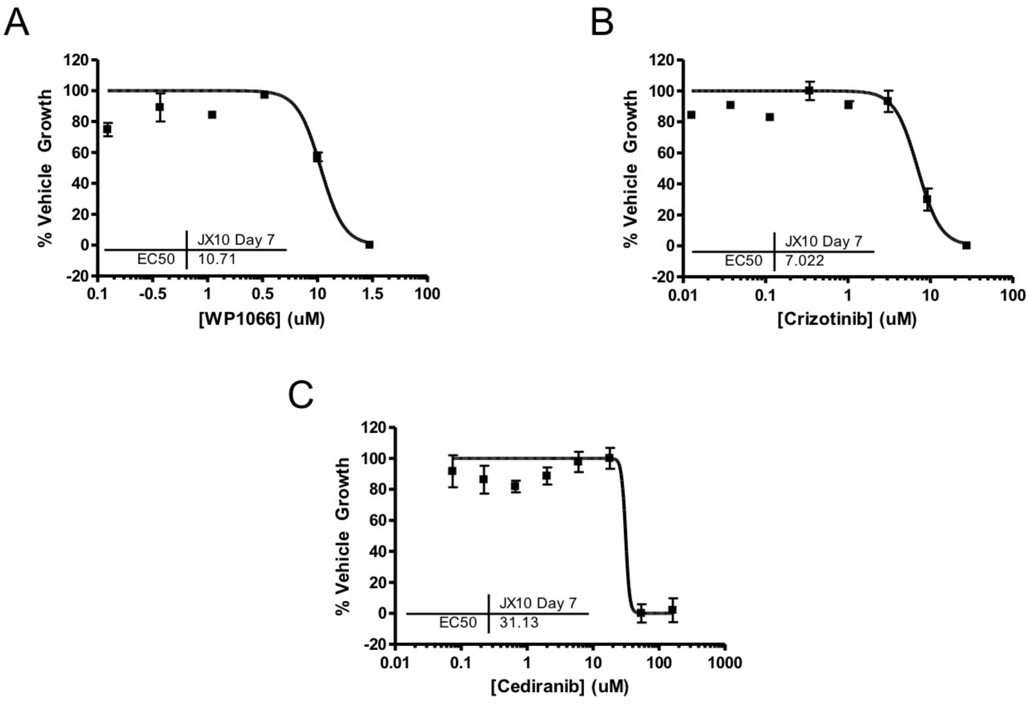

Supplemental Figure S3. Dose response curves for JX10UAB microtumors at Day 7. MTT absorbance relative to 0.5% DMSO (% Vehicle Control) is shown with non-linear regression curve fitted. Data shown for **(A)** WP1066, **(B)** Crizotinib, and **(C)** Cediranib.

Supplemental Table 1. Combination index values of WP1066, Selumetinib, Crizotinib, and Cediranib SMIs in combination for all xenolines at Day 7.

**Combination Dosing**	**XD456**	**X1066**	**JX10**	**JX22P**	**X1046**	**X101**
WP1066 $IC_{37.5}$ + Selumetinib $IC_{12.5}$	4.41	1.08	0.91	0.31	>10	NaN
WP1066 $IC_{37.5}$ + Crizotinib $IC_{12.5}$	2.83	1.23	1.44	0.47	>10	NaN
WP1066 $IC_{37.5}$ + Cediranib $IC_{12.5}$	9.58	1.40	1.43	0.63	1.20	NaN
WP1066 IC_{25} + Selumetinib IC_{25}	7.24	5.69	1.28	1.04	>10	NaN
WP1066 IC_{25} + Crizotinib IC_{25}	1.24	1.54	1.74	0.86	>10	NaN
WP1066 IC_{25} + Cediranib IC_{25}	2.47	1.69	3.95	0.96	>10	NaN
Selumetinib $IC_{37.5}$ + WP1066 $IC_{12.5}$	6.36	2.42	1.30	1.56	>10	NaN
Selumetinib $IC_{37.5}$ + Crizotinib $IC_{12.5}$	9.87	4.18	1.25	0.70	1.11	NaN
Selumetinib $IC_{37.5}$ + Cediranib $IC_{12.5}$	4.98	>10	7.83	1.00	>10	0.12
Selumetinib IC_{25} + Crizotinib IC_{25}	>10	1.82	0.81	0.22	9.31	NaN
Selumetinib IC_{25} + Cediranib IC_{25}	5.69	0.72	5.80	0.60	2.21	3.67
Crizotinib $IC_{37.5}$ + WP1066 $IC_{12.5}$	0.80	1.16	1.00	0.15	>10	NaN
Crizotinib $IC_{37.5}$ + Selumetinib $IC_{12.5}$	>10	0.97	0.57	0.08	3.73	NaN
Crizotinib $IC_{37.5}$ + Cediranib $IC_{12.5}$	>10	1.23	0.95	0.28	4.40	NaN
Crizotinib IC_{25} + Cediranib IC_{25}	0.85	1.06	1.17	0.61	0.08	NaN
Cediranib $IC_{37.5}$ + WP1066 $IC_{12.5}$	>10	1.11	1.00	1.30	3.34	NaN
Cediranib $IC_{37.5}$ + Selumetinib $IC_{12.5}$	2.90	0.71	0.75	0.67	3.31	1.75
Cediranib $IC_{37.5}$ + Crizotinib $IC_{12.5}$	0.76	0.96	0.93	0.79	0.09	NaN

Abbreviations: CI values, <1 is synergistic, =1 is additive, and >1 is antagonistic
